# An unusual presentation of longstanding atrophic Hashimoto’s thyroiditis: challenges in a resource-limited setting

**DOI:** 10.1530/EDM-24-0152

**Published:** 2025-11-11

**Authors:** Phoebe Wamalwa, Prisca Amolo

**Affiliations:** ^1^ Kenyatta University, Nairobi, Kenya; ^2^ Kenyatta National Hospital, Nairobi, Kenya

**Keywords:** unusual, presentation, longstanding, Hashimoto’s, thyroiditis

## Abstract

**Summary:**

Hypothyroidism, affecting approximately 5% of the population, often remains undiagnosed, more so in developing countries. We report a 12-year-old girl with longstanding, untreated hypothyroidism who initially presented with proteinuria. History revealed recurrent vomiting, poor appetite, lethargy, and neurodevelopmental delays. Examination showed short stature, ptosis, outer eyebrow hair loss, myxedematous facies, dry skin, and hypertrophied calves. Investigations confirmed severe primary hypothyroidism, Hashimoto’s thyroiditis, which most commonly presents between the ages of 45 and 55 years, with elevated thyroid autoantibodies and an atrophic thyroid gland. Additional findings were dyslipidemia and moderate proteinuria, both of which resolved with levothyroxine, which also improved growth. However, weight fluctuations persisted due to treatment non-adherence from financial challenges. This case illustrates a broad and atypical spectrum of complications arising from longstanding, untreated primary hypothyroidism. Awareness of such varied clinical manifestations is essential for timely diagnosis and appropriate management.

**Learning points:**

- Undiagnosed cases of hypothyroidism are more prevalent in poor-resource setups.
- Longstanding hypothyroidism can lead to varied presentations involving different organs, including the kidneys and gastrointestinal system, besides its profound negative effect on growth and brain development. Dyslipidemia and ptosis have been reported as well.
- Calf muscle hypertrophy, Kocher–Debré–Semelaigne syndrome, has been reported in longstanding hypothyroidism.

## Background

Hypothyroidism refers to a deficiency of circulating thyroid hormone due to reduced glandular activity. It may be classified as either primary or secondary (central) hypothyroidism (1). Primary hypothyroidism results from intrinsic dysfunction of the thyroid gland, either congenital (e.g. dyshormonogenesis, thyroid agenesis, or athyreosis) or acquired, such as autoimmune thyroiditis. Iodine deficiency also remains a prevalent cause of primary hypothyroidism globally. In contrast, secondary hypothyroidism arises from hypothalamic or pituitary dysfunction (1).

Hashimoto’s thyroiditis is the most common cause of hypothyroidism in developed countries (2). It is an autoimmune disorder characterized by lymphocytic infiltration of the thyroid gland, leading to progressive fibrosis and follicular cell destruction through both T-cell–mediated and antibody-mediated mechanisms. The disease predominantly affects females, with a female-to-male ratio of 7–10:1 (2), and most commonly presents between the ages of 45 and 55 years. Affected individuals typically harbor multiple antithyroid antibodies: thyroid peroxidase antibodies (present in >90%), thyroglobulin antibodies (50–80%), and variably thyroid-stimulating hormone (TSH) receptor-blocking antibodies (2, 3). Thyrotropin receptor antibodies (TRAb) target the TSH receptor and are clinically relevant in both hyperthyroidism and autoimmune thyroid disease. While thyroid peroxidase (TPO) and thyroglobulin (Tg) antibodies are more strongly associated with Hashimoto’s thyroiditis, TRAb are typically present in Graves’ disease, though they may occasionally be detected in Hashimoto’s (4).

Untreated chronic hypothyroidism in children can lead to impaired growth, cognitive decline, memory deficits, and delayed speech. Rare complications also occur, including Kocher–Debré–Semelaigne syndrome (KDSS), a condition characterized by muscular pseudohypertrophy associated with longstanding moderate to severe hypothyroidism, typically between 3 and 10 years of age (5, 6). The pathogenesis of muscle changes remains unclear but is thought to reflect prolonged hypothyroid effects on muscle fibers, with nonspecific histological findings. The clinical severity of hypothyroidism generally correlates with the degree of pseudohypertrophy and cramps. Both hypothyroid symptoms and muscular changes are reversible with thyroxine therapy (5, 6).

## Case report

A 12-year-old girl from Kajiado County, Kenya, was referred from the nephrology clinic to the pediatric endocrine clinic for further evaluation due to concerns about her abnormal physical appearance. She had initially presented with generalized body swelling to a private facility 1 week earlier, where urinalysis revealed moderate proteinuria. She was empirically started on prednisolone (10 mg once daily) for suspected nephrotic syndrome. At the nephrology clinic, further evaluation confirmed 2+ proteinuria on urinalysis with normal renal function. There was no history of hematuria, fever, sore throat, or joint pain.

Historical review revealed generalized inactivity described as ‘lacking energy’, persistent vomiting since birth (reduced at 6 months of age but still episodic, especially after feeding), reduced appetite, and exclusive feeding on small portions. Bowel movements were reported as normal, with no polyuria or polydipsia. She slept for about 10 h nightly. Notably, she had memory impairment, poor school performance, and delayed speech development since the age of two.

Perinatal history revealed she was born at term via spontaneous vaginal delivery with a birth weight of 2.5 kg. She cried the day after birth, with unclear documentation of resuscitation. Breastfeeding commenced on day 1 but with weak suckling. There were no neonatal seizures, hypoglycemia, or jaundice. Milestones were significantly delayed: she sat at 1 year, walked at 2 years, and began speaking at 4 years. She was exclusively breastfed for about 3 months, after which cow’s milk was introduced. Her diet was chronically deficient in vegetables, and poor appetite persisted through early childhood.

She is the second-born of five siblings. The first-born male (16 years) is tall and performs averagely in school. The third-born male (10 years) and fourth-born male (8 years) are both taller than the index case and perform well academically. The fifth child is a 2-year-old male. The family is pastoralists, and there is no reported family history of thyroid disease, diabetes, or hypertension.

### Examination findings

**Figure 1:**
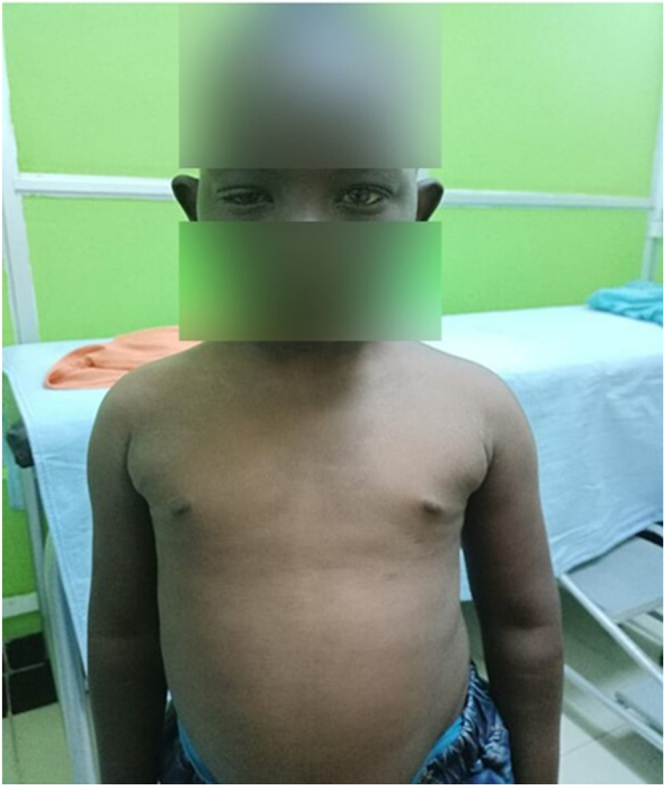
A short and small-for-age girl is shown here with generalized hard/dry skin, non-pitting edema, myxedematous face, right eye ptosis, a flat affect, absent goiter, and distended abdomen.

She appeared stunted and underweight for her age. She had generalized non-pitting edema, dry and coarse skin, hair loss in the lateral eyebrows, lower eyelid edema, and a flat affect. Additional features included myxedematous facies, coarse facial features, large ears, right eye ptosis, and no goiter (Fig. 1). She was not pale.

**Figure 2:**
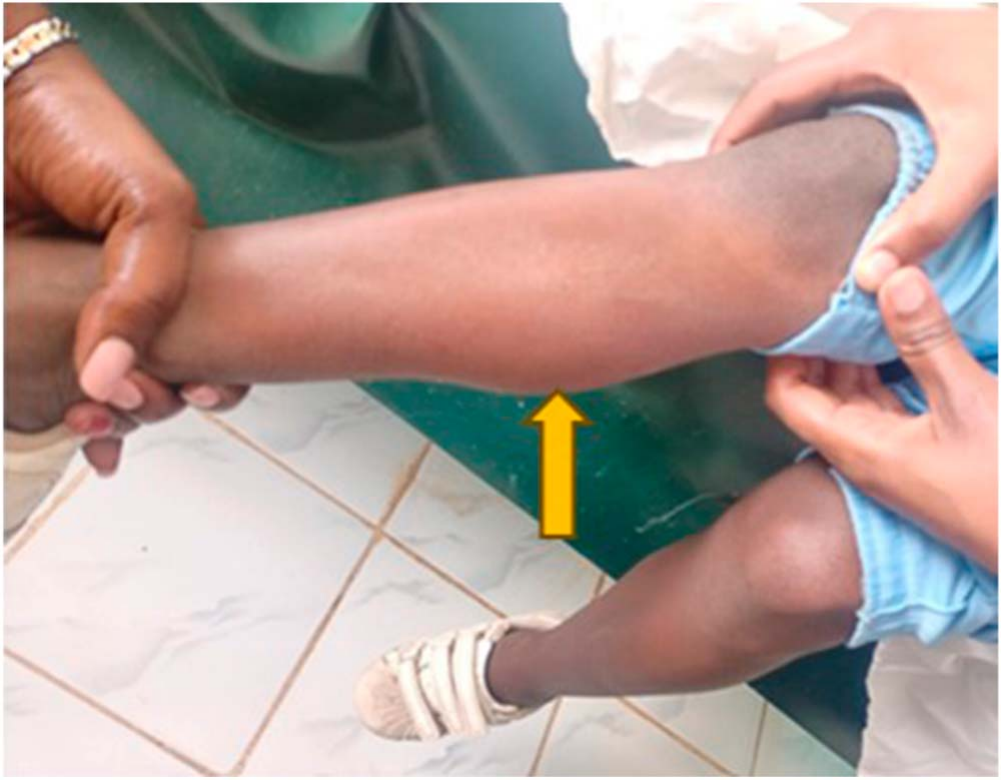
Right calf muscle pseudohypertrophy (Kocher–Debré–Semelaigne syndrome), indicated by the yellow arrow.

Her vital signs were as follows: pulse rate 64 bpm (regular), SpO_2_ 99%, and blood pressure 96/67 mmHg. Respiratory and cardiovascular examinations were normal, with no murmurs. Abdominal examination revealed moderate distension, no organomegaly, and reduced bowel sounds. Calf muscle hypertrophy was observed, consistent with Kocher–Debré–Semelaigne syndrome (Fig. 2). Genitalia were normal female. Pubertal staging was Tanner B1, P1, A0. No other deformities were noted.

Anthropometric measurements were:

Height: 120.5 cm (−4.4 SDS).

Weight: 24 kg (−3.45 SDS).

Head circumference: 48 cm (−3.5 SDS).

**Figure 3:**
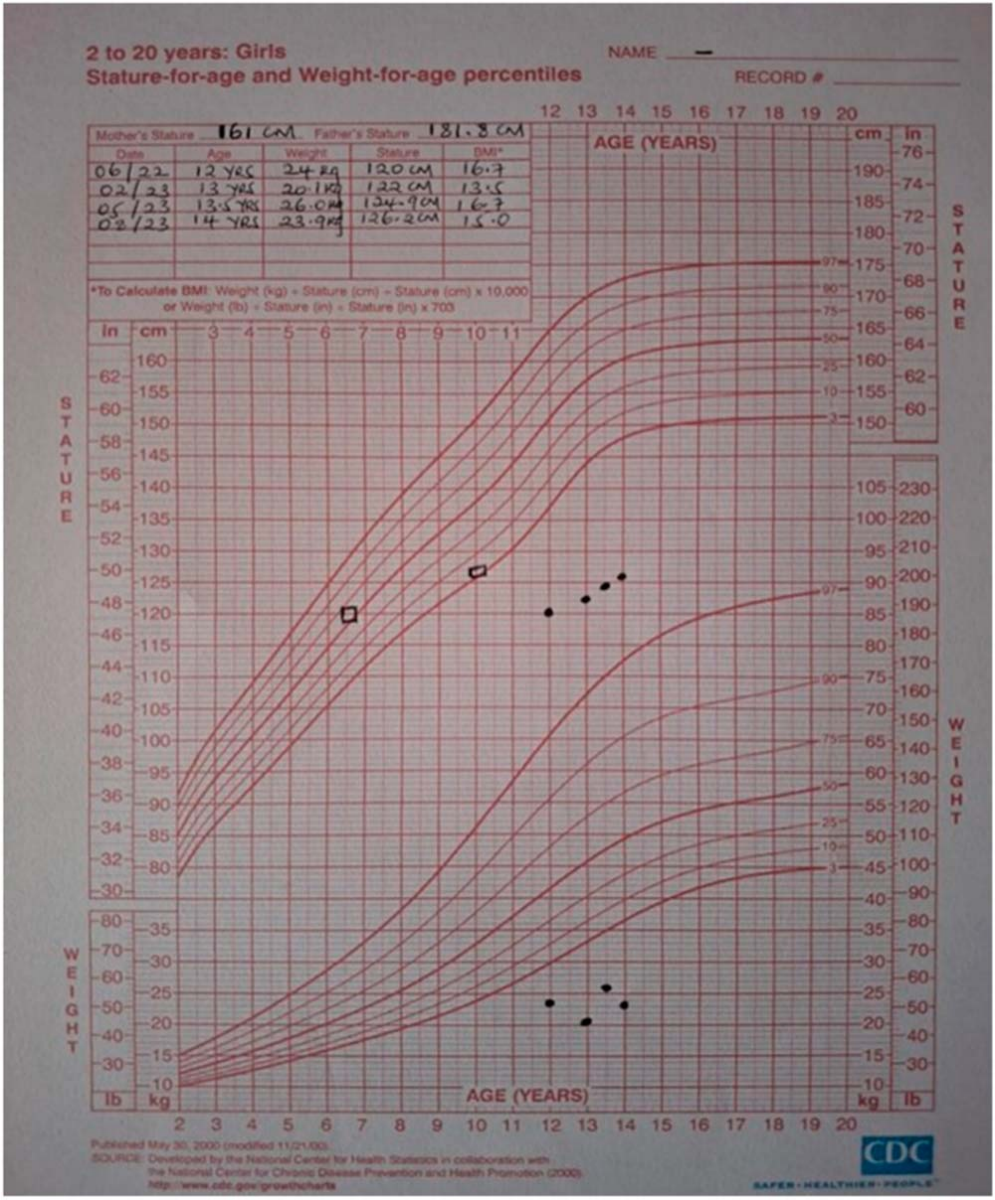
Growth chart showing fluctuation of weight, sustained height velocity, with delayed bone age.

Mid-parental height was estimated at 165 cm (mother: 161 cm, 81 kg; father: 181.8 cm, 70 kg), (Fig. 3).

## Investigation

Thyroid function tests confirmed severe primary hypothyroidism. Lipid profile revealed dyslipidemia, and urinalysis confirmed moderate proteinuria (Table 1). Thyroid peroxidase and thyroglobulin antibodies were elevated; thyroglobulin levels were severely reduced. Thyroid receptor and acetylcholine receptor antibodies were negative (Table 2). All assays were performed at PathCare, an ISO 15189-accredited laboratory, following standard operating procedures with internal and external quality controls. Venous blood was collected in plain tubes, centrifuged within 2 h, and serum analyzed the same day or stored at −20°C for longer storage to facilitate shipping to a central reference laboratory for specialized receptor antibody testing. Thyroid peroxidase antibodies (TPOAb), thyroglobulin antibodies (TgAb), and TSH receptor antibodies (TRAb) were quantified using automated electrochemiluminescent immunoassays traceable to international standards. Thyroid function tests (TSH, free T4, free T3) were measured by third-generation ECLIA with checks for biotin or heterophile interference. Lipid profiles (total cholesterol, HDL-C, triglycerides, LDL-C, calculated by Friedewald) were analyzed enzymatically. Acetylcholine receptor antibodies were measured by a validated high-specificity immunoassay (radioimmunoassay). Calibration, lot verification, and age/sex-specific reference intervals were as per PathCare protocols, and results were reported in SI units, with flagged abnormal values. Urinalysis was performed on a freshly voided midstream urine sample using a standard dipstick method, which revealed the presence of proteinuria.

**Table 1:** Laboratory tests: lipid profile, thyroid function tests, urinalysis. Abnormal values are presented in bold.

Tests	Reference	Baseline	At 2 months	At 5 months	At 24 months
TSH (IU/L)	0.25–5	**>100**	**0.05**	1.63	**>100**
FT4 (pmol/L)	10.6–19.4	**3**	**38.12**	**26.93**	**3.6**
FT3 (pmol/L)	4–8.3	**1.34**	-	-	**0.85**
LDL (mmol/L)	<2.8	**3.28**	-	-	
Cholesterol (mmol/L)	3.5–5.6	**6.82**	-	**5.4**	**7.15**
HDL	0.8–2	1.77	-	**1.62**	**1.38**
Triglyceride (mmol/L)	0.8–2	**3.69**	-	**2.98**	**6.8**
HDL/LDL ratio	0.3–2	0.5	-	-	-
Total/HDL ratio	<3.5	**3.9**	-		
Proteinuria		**2+**	**Nil**		**Trace**

TSH, thyroid-stimulating hormone; FT4, free thyroxine; FT3, free triiodothyronine; LDL, low-density lipoproteins; HDL, high-density lipoproteins.

**Table 2:** Laboratory tests: thyroid and acetylcholine receptor antibodies.

Test	Results	Reference
TG, μg/L	<0.1	<78
TG-ab, IU/mL	885.0	<4.1
TPO-ab, IU/mL	>1,000	0.0–5.6
TRAB, U/L	<0.8	<1.75
AchR 11F	Negative	
AchR Fetal 11F	Negative	
AchR ab Elisa, nmol/L	<0.11	>0.50
MuSK 11F	Negative	

TG, thyroglobulin; TG ab, thyroglobulin antibody; TPO, thyroid peroxidase antibodies; Trab, thyroid receptor antibody; AchR, acetylcholine receptor; MuSK, muscle-specific kinase antibodies. (Up to 10% of MG patients may be seronegative).

**Figure 4:**
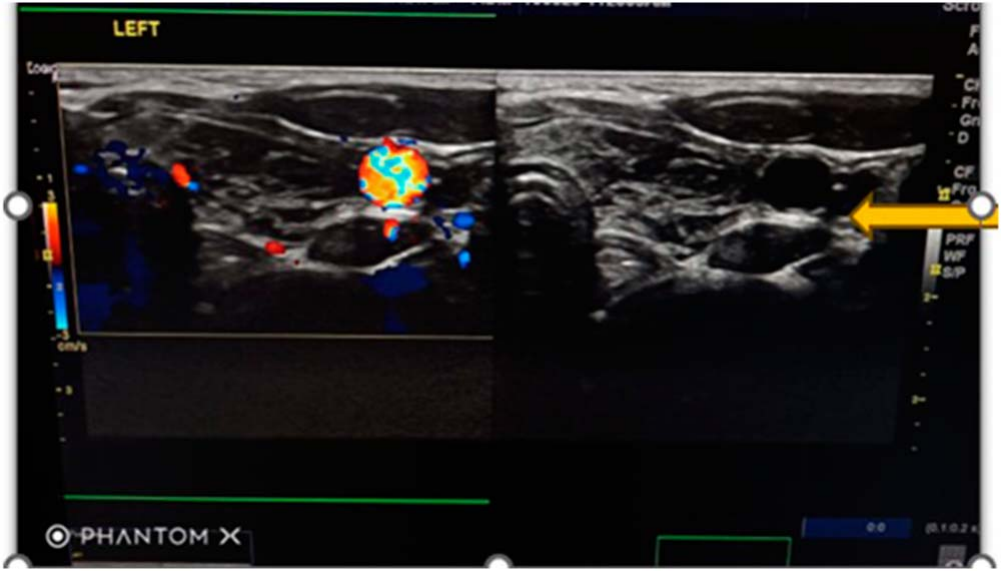
Thyroid ultrasound showing atrophic thyroid gland, normal neck vasculature, yellow arrow.

Thyroid ultrasound showed marked atrophy of the thyroid gland, with normal neck vasculature (Fig. 4). Thyroid ultrasonography was performed using a high-frequency (7.5–12 MHz) linear transducer with the patient in the supine position and the neck slightly extended. Both thyroid lobes and the isthmus were systematically examined in longitudinal and transverse planes to assess gland size, echotexture, and margins. Color Doppler was applied to evaluate vascularity, and adjacent cervical structures and vasculature were reviewed. The scan demonstrated an atrophic thyroid gland with preserved and normal-appearing neck vasculature.

**Figure 5:**
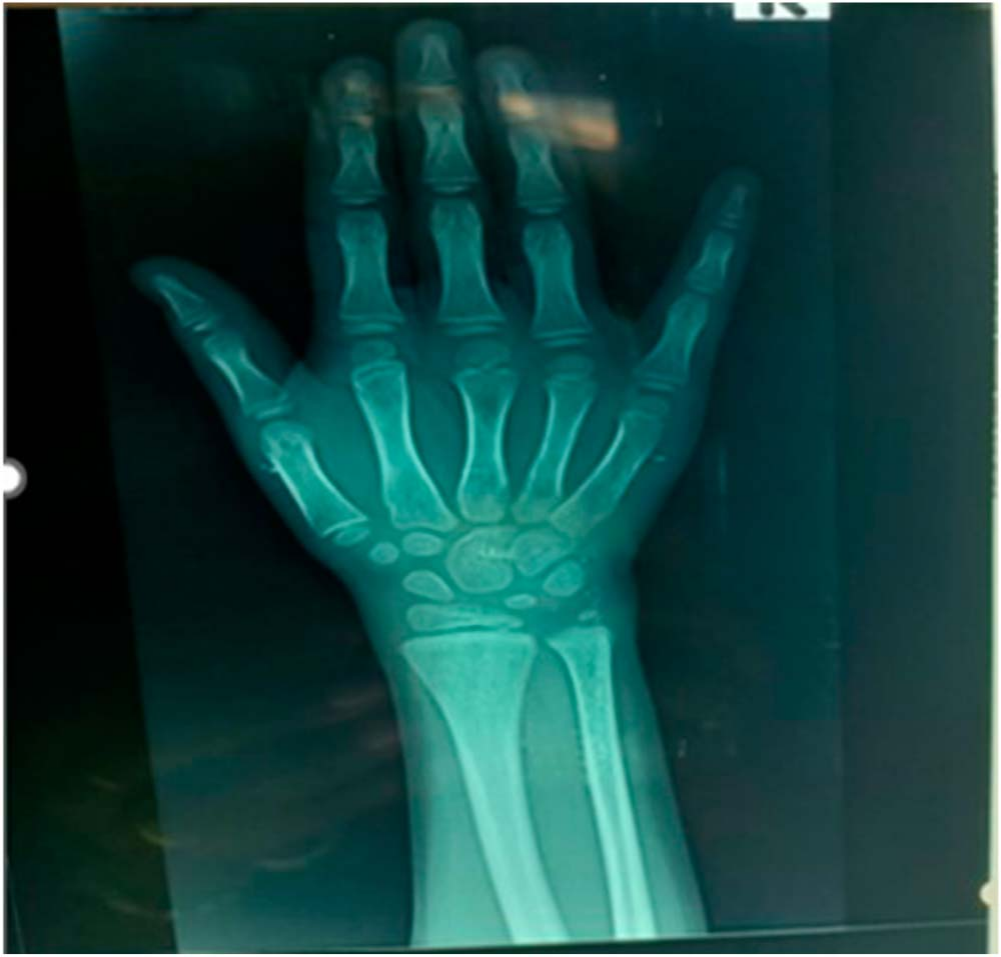
The bone age showing delay at 6 years 10 months at a chronological age of 12 years.

Bone age was significantly delayed at 6 years and 10 months compared to a chronological age of 12 years (Fig. 5). Bone age assessment was performed using a plain radiograph of the left hand and wrist. The radiograph was compared with standard reference images in the Greulich and Pyle atlas, and the corresponding bone age was determined. The derived bone age was then plotted against the patient’s chronological age on a standardized growth chart to illustrate the degree of delay. Due to limited resources, further laboratory testing required fundraising and external sample referral.

## Treatment

The patient was initiated on levothyroxine at 2.5 μg/kg/day (50 μg daily), with plans for dose titration based on follow-up thyroid function tests. Repeat lipid profile was scheduled for 3 months; however, lipid-lowering therapy was not initiated. Growth and pubertal development were to be monitored at each clinic visit. The levothyroxine dose was gradually increased, leading to transient hyperthyroidism, necessitating dose reduction to initial levels to maintain a euthyroid state.

## Outcome and follow-up

**Figure 6:**
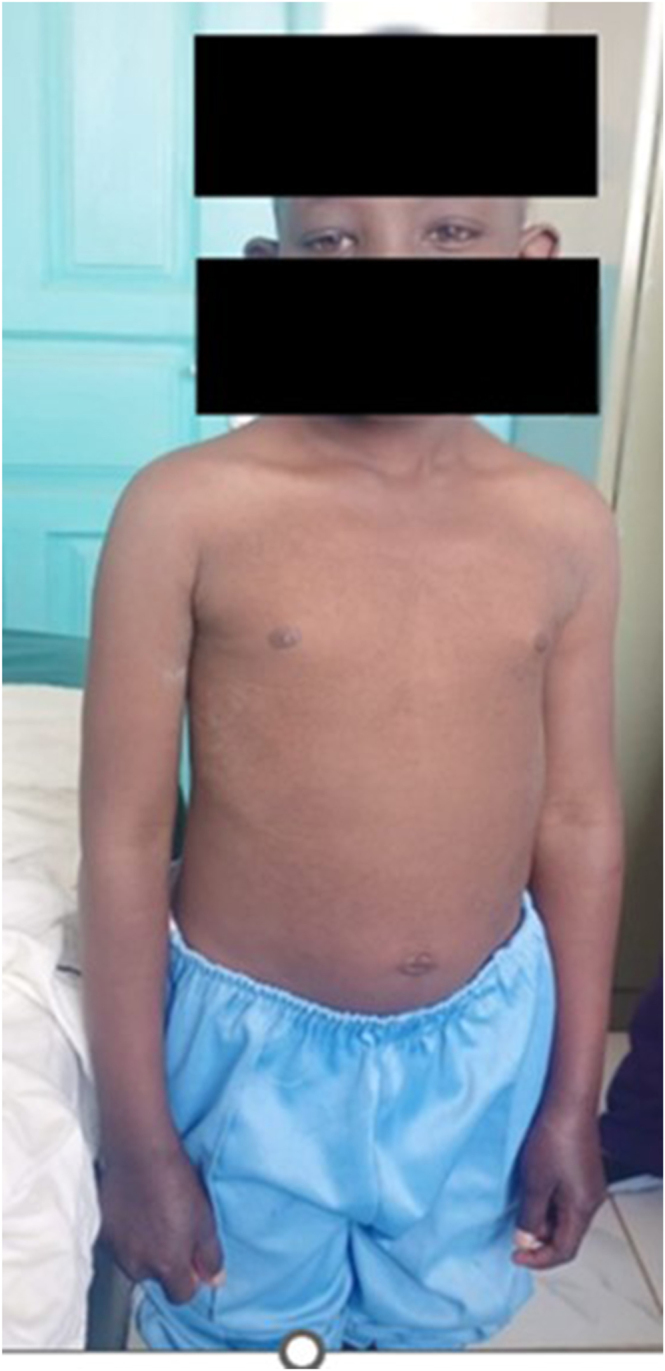
The patient after 2 years of treatment. She appears taller, lean, with dry skin; ptosis still present but less marked.

After 6 weeks of levothyroxine therapy, proteinuria resolved, with steroid treatment having been discontinued after only 2 weeks of initiation. Lipid profile normalized after 5 months of treatment for hypothyroidism. The child’s growth velocity improved to 8.4 cm/year. Bone age advanced from 6 years 10 months (at age 12) to 10 years (at age 14) over a 2-year period. She showed significant improvement in weight and fluid status (Fig. 6).

However, after 18 months of consistent follow-up, the patient became noncompliant and was lost to follow-up. Upon re-presentation, she had developed signs of myxedema again, with weight gain, recurrence of dyslipidemia, and trace proteinuria.

## Discussion

This patient presented with multiple complications of prolonged untreated hypothyroidism, including cognitive impairment, growth retardation, memory and speech delays, recurrent vomiting, ptosis, dyslipidemia, Kocher–Debré–Semelaigne syndrome (KDSS), and proteinuria. This discussion will focus on the latter five manifestations.

Vomiting is a recognized but uncommon manifestation of hypothyroidism. Vidwans *et al.* described two such cases, attributing the symptom to gastrointestinal dysmotility secondary to thyroid hormone deficiency (7). Pathophysiological mechanisms include reduced gastrointestinal electrical and motor activity, decreased sigmoid and rectal contractions, delayed gastric emptying, reduced lower esophageal sphincter pressure and contractility, and pseudo-obstruction due to sporadic non-propagated contractions in the small intestine (7). In addition, vomiting in autoimmune thyroiditis may be associated with coexisting celiac disease (3); however, testing for celiac antibodies was not performed in this case. Our patient had recurrent vomiting since infancy, which resolved with the initiation of levothyroxine, suggesting a direct link to hypothyroidism.

Proteinuria in hypothyroidism may be a consequence of chronic disease, a contributing factor to thyroid dysfunction, or part of the broader autoimmune spectrum. Ranga *et al.* reported reversible proteinuria in patients with hypothyroidism (8). Thyroid hormones influence renal development, glomerular filtration rate, nephron function, and fluid-electrolyte balance, both directly and via cardiovascular effects (5). Conversely, the kidneys contribute to thyroid hormone metabolism and clearance (9). Renal disease, particularly nephrotic syndrome, can induce hypothyroidism through urinary losses of thyroid-binding globulin, albumin, and iodine, leading to reduced T3/T4 levels and impaired hormone synthesis (9). Hypoproteinemia may further impair thyroid hormone production by limiting substrate availability and inducing gastrointestinal mucosal edema (9). In this case, proteinuria resolved rapidly with levothyroxine and brief corticosteroid therapy, with normal renal ultrasound findings, making autoimmune nephropathy unlikely. Renal biopsy was not performed due to financial constraints.

Dyslipidemia is a well-established complication of hypothyroidism. Devina *et al.* described elevated total cholesterol (TC) and low-density lipoprotein cholesterol (LDL-C) due to decreased LDL receptor activity, impaired lipoprotein lipase (LPL) function, and reduced hepatic lipase activity, resulting in elevated triglycerides (TG), very-low-density lipoprotein (VLDL), and HDL-C levels (10). Cholesteryl ester transfer protein (CETP) deficiency contributes to high HDL-C by impairing cholesteryl ester transfer to VLDL (11). Our patient had elevated TC, LDL-C, TG, and borderline high HDL-C, with normalization upon levothyroxine therapy. However, dyslipidemia recurred upon treatment interruption with levothyroxine, confirming a direct relationship with hypothyroidism.

Ptosis is a rare manifestation of hypothyroidism. Chitra *et al.* linked it to hypothyroid myopathy, which may mimic polymyositis with proximal muscle weakness and elevated creatine kinase (12). Additional mechanisms include glycosaminoglycan-mediated compression of the third cranial nerve and associated vascular compromise, which may necessitate surgical intervention (12). Myasthenia gravis, though uncommon, may co-occur with autoimmune thyroid disease and contribute to ptosis via anti-acetylcholine receptor (AChR) antibodies. While AChR and muscle kinase antibodies were negative in this patient, seronegativity is common in ocular myasthenia (12). Pituitary adenomas invading the cavernous sinus may also cause ptosis; however, MRI was not performed. The patient demonstrated partial response to levothyroxine, though mild ptosis persisted.

Kocher–Debré–Semelaigne syndrome (KDSS), characterized by calf muscle pseudohypertrophy, is a rare pediatric manifestation of longstanding untreated hypothyroidism. The underlying pathology involves impaired carbohydrate metabolism and glycogen accumulation, along with mucopolysaccharide deposition and increased connective tissue (13). Electromyography, often normal or showing myopathic changes, and histology, which may reveal fiber diameter variation, metachromasia, segmental degeneration, or lymphatic infiltration but can also be normal (5, 6), were not performed due to resource constraints. However, clinical improvement in muscle bulk was noted following levothyroxine initiation.

Notably, the patient demonstrated accelerated bone maturation during treatment, consistent with findings from other reports (8). This, along with the onset of puberty, may compromise final height potential despite catch-up growth.

## Conclusion

This case illustrates a broad and atypical spectrum of complications arising from longstanding untreated primary hypothyroidism. Awareness of such varied clinical manifestations is essential for timely diagnosis and appropriate management.

## Declaration of interest

The authors declare that there is no conflict of interest that could be perceived as prejudicing the impartiality of the work reported.

## Funding

This research did not receive any specific grant from any funding agency in the public, commercial, or not-for-profit sector.

## Patient consent

Written informed consent for publication of the clinical details and/or clinical images was obtained from the parent of the patient.

## Author contribution statement

PA, the co-author, was consulted on patient management. She also took part in the review and editing of the case report. All authors approved the article for publication.
